# Diagnosis of hemidiaphragm paralysis: refine ultrasound criteria

**DOI:** 10.3389/fmed.2024.1416520

**Published:** 2024-05-23

**Authors:** Alain Boussuges, Alex Fourdrain, Marc Leone, Geoffrey Brioude, Amelie Menard, Laurent Zieleskiewicz, Stephane Delliaux, Marion Gouitaa, Hervé Dutau, Fabienne Brégeon

**Affiliations:** ^1^Centre de Recherche en Cardio-Vasculaire et Nutrition, C2VN (Université Aix Marseille, INSERM 1263, INRAE 1260), Faculté de Médecine, Marseille, France; ^2^Laboratoire d’Exploration Fonctionnelle Respiratoire, Hôpital Nord, Assistance Publique des Hôpitaux de Marseille, Marseille, France; ^3^Département de Chirurgie Thoracique, Hôpital Nord, Assistance Publique des Hôpitaux de Marseille, Marseille, France; ^4^Service d’Anesthésie et Réanimation, Hôpital Nord, Assistance Publique des Hôpitaux de Marseille, Marseille, France; ^5^Service de Médecine Interne, Unité Post COVID, Hôpital Nord, Assistance Publique des Hôpitaux de Marseille, Marseille, France; ^6^Département des Maladies Respiratoires et Transplantation Pulmonaire, Hôpital Nord, Assistance Publique des Hôpitaux de Marseille, Marseille, France; ^7^Unité d’Appui à la recherche (HIPE), Aix-Marseille Université, CNRS, Université de Toulon, Institut Paoli-Calmettes, Marseille, France

**Keywords:** chest ultrasonography, diaphragm excursion, diaphragm thickness, thickening fraction, respiratory failure

## Abstract

**Background:**

Ultrasound has demonstrated its interest in the analysis of diaphragm function in patients with respiratory failure. The criteria used to diagnose hemidiaphragm paralysis are not well defined.

**Methods:**

The aim of this observational retrospective study was to describe the ultrasound findings in 103 patients with diaphragm paralysis, previously diagnosed by conventional methods after various circumstances such as trauma or surgery. The ultrasound study included the recording of excursions of both diaphragmatic domes and the measurement of inspiratory thickening.

**Results:**

On paralyzed hemidiaphragm, thickening was less than 20% in all patients during deep inspiration. Thinning was recorded in 53% of cases. In some cases, the recording of the thickening could be difficult. The study of motion during voluntary sniffing reported a paradoxical excursion in all but one patient. During quiet breathing, an absence of movement or a paradoxical displacement was observed. During deep inspiration, a paradoxical motion at the beginning of inspiration followed by a reestablishment of movement in the cranio-caudal direction was seen in 82% of cases. In some patients, there was a lack of movement followed, after an average delay of 0.4 s, by a cranio-caudal excursion. Finally, in 4 patients no displacement was recorded. Evidence of hyperactivity (increased inspiratory thickening and excursion) of contralateral non-paralyzed hemidiaphragm was observed.

**Conclusion:**

To accurately detect hemidiaphragm paralysis, it would be interesting to combine the ultrasound study of diaphragm excursion and thickening. The different profiles reported by our study must be known to avoid misinterpretation.

## Introduction

Hemidiaphragm paralysis is widely underestimated and can cause dyspnea or delayed recovery after respiratory distress or thoracic surgery. The interest of the assessment of diaphragm function by ultrasound at admission to the emergency room or intensive care unit has recently been highlighted (1–4).

In trauma patients, diaphragmatic rupture may be secondary to penetrating or blunt chest and abdominal trauma (5, 6). In severe trauma patients, impaired respiratory function can be attributed to the severity of the shock or to lung damage. Therefore, diaphragm dysfunction can be difficult to identify, and diagnosis is often delayed. Diaphragmatic dysfunction can also be secondary to phrenic nerve injury. The lesion of the nerve along its path from the cervical vertebra to the diaphragmatic dome may be secondary to the trauma or to a cervical or thoracic surgery (7–9). The value of point-of-care ultrasound for detecting diaphragm dysfunction after thoracic surgery was recently underlined as diaphragm dysfunction was associated with postoperative lung complications (10).

In medical patients suffering from respiratory failure, diaphragm dysfunction can result from several causes such as brain lesions, neuromuscular diseases or impairment in phrenic nerve conduction (11). In stroke patients, it has been reported that impairment of the respiratory centers leads to a decrease in diaphragmatic excursion (12). In neuromuscular diseases, the degradation of diaphragmatic function is correlated with the course of the disease. In acute infectious diseases, diaphragm function may be impaired by phrenic nerve damage. For example, in patients with COVID-19, diaphragm dysfunction was observed in 10% of patients, including 2 cases of hemidiaphragm paralysis (13). In patients with pulmonary disease the severity of the respiratory status can be increased by pre-existing diaphragm dysfunction. Lastly, in critically ill patients, impaired diaphragmatic function is frequently involved in weaning failure (14).

The most serious form of hemidiaphragm dysfunction is the complete paralysis. Hemidiaphragm paralysis is most frequently well tolerated in previously healthy subjects. In contrast, tolerance is bad in patients with comorbidities such as obesity, and cardiac or respiratory disorders (15, 16). In these patients and in case of bilateral diaphragm dysfunction, ventilatory mechanical support may be necessary (17–19).

Therefore, analysis of diaphragm function in a risk situation is important for early diagnosis. To detect hemidiaphragm paralysis on admission to emergency or intensive care unit, ultrasound is the most appropriate tool because it is easy to learn and can be performed at the bedside (20). For a detailed analysis of diaphragm function by ultrasound, it is interesting to study the excursion and thickening of the diaphragm. Diaphragm excursion is sensitive to changes in respiratory patterns and is related to the diaphragm volume generation capacity (21, 22). In addition, the measurement of diaphragmatic thickening makes it possible to estimate the contribution of both hemidiaphragms to the work of breathing (23).

In patients with hemidiaphragm paralysis, M-mode ultrasound can be informative by detecting abnormal motion of the hemidiaphragm and a lack of excursion during inspiration (24, 25). Measurement of inspiratory thickening has also demonstrated its interest. A lack of inspiratory thickening is in favor of hemidiaphragm paralysis (26).

Nevertheless, the criteria used to diagnose hemidiaphragm paralysis by ultrasound were determined from case reports (27, 28) or small series of adult patients (24–26). It would be important for an accurate description of ultrasound criteria to study a large population of patients with a recognized diagnosis.

The objective of this observational retrospective study was to report a complete description of ultrasound results, including excursion and diaphragm thickness analysis, from the study of a large population of patients with a prior and recognized diagnosis of diaphragm paralysis.

## Methods

This was a retrospective observational study carried out at the North Hospital in Marseilles, France. This study fell within the French legal framework of non-interventional research. It consisted in an analysis of data collected during standard clinical care and was classified as non-involving human subjects. The research was registered under the number RGPD PADS-20-252 in the Marseilles hospital institution. Data were collected from ultrasound consultations performed in the lung function laboratory. Medical charts were used to identify cases of diaphragm paralysis and subjects with normal lung function. In the medical consultation unit, all patients received a notice of information and non-objection in accordance with French law. The study adhered to the ethical standards outlined in the 2008 declaration of Helsinki.

### Populations studied

#### Patients with hemidiaphragm paralysis

This retrospective study enrolled patients from a consultation designed to study diaphragm function by ultrasonography. The included patients were referred to the ultrasound consultation from January 2018 to October 2023. Diagnosis of diaphragmatic paralysis was established before ultrasound, the more frequently by another team, sometimes from another hospital. It was often suggested by chest radiography, which revealed an elevation of the affected hemidiaphragm. It has also been supported by functional evaluations, such as pulmonary function tests (PFTs) that reported a decreased maximal mouth inspiratory pressure, decreased nasal inspiratory pressure during sniff maneuver. Other investigations included fluoroscopy and diaphragm electromyography (29). In Marseille’s hospitals, the compound motor action potentials of the two hemidiaphragms are most frequently recorded with chest surface electrodes following phrenic nerve stimulation (electric or magnetic) in the neck. In patients with hemidiaphragm paralysis, electromyography exhibited a failure of diaphragmatic response to phrenic nerve stimulation on the paralyzed side. In some patients, paralysis was recognized from the clinical context (surgery with section of the phrenic nerve for example). The time between ultrasound and other examinations was as short as possible.

It is known that pleural effusion can impair diaphragmatic motion, leading in some cases to an appearance of hemidiaphragm paralysis. Therefore, patients with significant pleural effusion on the paralyzed side were not included in the study.

#### Subjects with no pulmonary disease

Subjects with normal lung function were recruited from the consultation of the Pulmonary Function Testing unit of the North Hospital in Marseilles conducted from June 2020 to October 2023. Subjects were considered to have no pulmonary disease when they did not have a history of respiratory problems, normal chest imaging, no clinical disorders at the time of examination, and a normal lung function test.

### Ultrasound study

The ultrasonographic examinations were carried out by an experienced investigator (more than 1,000 diaphragm ultrasound examinations performed prior to the beginning of the study). All subjects were investigated in a seated position. The study used a commercially available ultrasound machine (Vivid S60N, GE Medical System, Milwaukee, Wl, United States) connected to a 1.5–3 MHz transducer array (3Sc probe) for excursion measurements and to a linear vascular transducer (9L probe) for thickness measurements. All ultrasound studies had been saved for subsequent blind analysis. To strengthen the accuracy of the results, measurements of ultrasound parameters were averaged from at least three different breathing cycles. To perform measurements at the right time and easily detect paradoxical motions, we used a system developed in our laboratory that can be connected to the ultrasound machine (30). Subjects were breathing through a device that measured inspiratory and expiratory volumes (31). The device incorporated in the turbine support, 3 sensors (1 infrared LED and 2 infrared phototransistors) to detect the changes in the respiratory cycle. Electronic signals corresponding to the beginning of the breathing time, i.e., inspiration and expiration, were sent to the ultrasound screen via ECG cables. To record the beginning of inspiration and expiration, a voltage pulse was sent at the time of the change of direction of rotation of the turbine. The voltage pulse was higher at the beginning of inspiration, which led to a greater amplitude of the signal on the ultrasound screen (see figures).

#### Assessment of diaphragm excursion

The patients were studied on both sides and diaphragm excursions were assessed by M-mode ultrasonography using a previously published method (32). A subcostal or low intercostal probe position was chosen between the midclavicular and posterior axillary lines in order to obtain the best imaging of both hemidiaphragmatic domes in two-dimensional mode (B-mode). When the visualization of the hemidiaphragm was considered good, anatomical M-mode was used to an accurate perpendicular approach. Diaphragm displacement was assessed while breathing at tidal volume (quiet breathing) and during voluntary sniffing. In addition, diaphragm excursions were measured during deep inspiration (from functional residual capacity to total lung capacity). The diaphragm inspiratory excursions were measured by placing the first caliper at the foot of the inspiration slope on the diaphragmatic echo line and placing the second caliper at the top of the curve. For deep inspiration, several maneuvers were recorded, and the maximal excursion, i.e., the greatest distance between the baseline and the apex, was retained. All the excursions (negative or positive) were measured. Furthermore, thanks to the transfer of the breathing signal to the ultrasound screen it was possible to measure the delay between the excursion and the beginning of the inspiratory effort.

#### Assessment of diaphragm thickening

**Figure 1:**
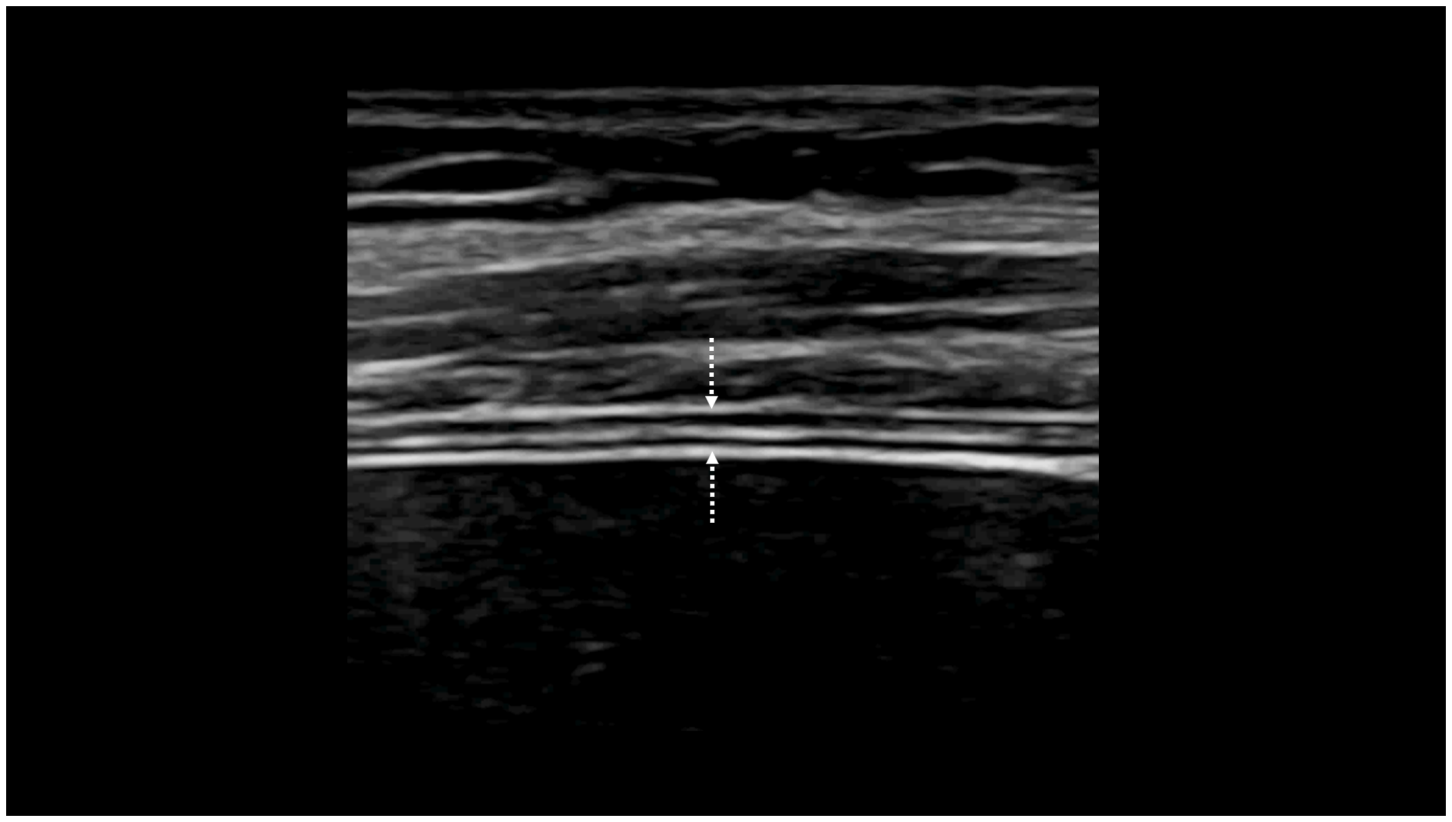
Visualization of the hemidiaphragm at the zone of apposition of the diaphragm to the rib cage: measurement of the thickness from the pleural line (downwards arrow) to the peritoneal line (upwards arrow).

Diaphragmatic thickness was assessed by B-mode ultrasonography using a previously published method (33). Both hemidiaphragms were visualized at the zone of apposition, and the probe was placed below the phrenico-costal sinus near the anterior or mid-axillary line at the eighth or ninth intercostal space. The diaphragm was identified as a three-layered structure with two parallel echogenic lines, the diaphragmatic pleura and the peritoneal fascia, enclosing the hypoechoic diaphragmatic muscle (Figure 1). A third hyperechoic line was frequently observed in the middle of the non-echogenic layer, considered to be the fibrous layer in the center of the diaphragm. The intercostal space that provided the best visualization of the diaphragm was chosen and the probe was positioned so that the two lines delimiting the diaphragm were parallel. The thickness of each hemidiaphragm was directly measured from the frozen B-mode images. The diaphragm thickness was measured as the distance between the pleural membrane and the peritoneal membrane, without including these lines in the measurement (34). The measurements were made at end-expiratory time (when the lung was filled at the functional residual capacity), at the end of inspiration during quiet breathing at tidal volume, and after deep inspiration up to total lung capacity (TLC). The percentage of thickening, i.e., the difference between end-inspiration and end-expiration thickness divided by the thickness at end-expiration, was determined for both hemidiaphragms during quiet breathing (inspiratory thickening) and during deep inspiration (thickening fraction).

### Lung function test

The results of pulmonary function tests (PFTs) and medical history were collected from patient’s medical charts. PFTs were performed according to the ERS/ATS standards (35). PFTs included spirometry and body plethysmography to measure expiratory volume in 1 s (FEV_1.0_), forced vital capacity (FVC), slow vital capacity (SVC), residual volume (RV) and total lung capacity (TLC) (PFT MasterLab Jaeger plethysmograph, Bunnik, The Netherlands). The criteria for classifying the pulmonary function test as normal were SVC, FVC and FEV_1.0_ higher than the lower limit of normal (LLN) for the reference population (36). The lung functional defects were screened according to ATS/ERS definitions (37). A restrictive pattern was attested in presence of reduced (lower than lower limit of normal) TLC, FVC and FEV_1.0_ with a normal or increased FEV_1.0_-to-FVC ratio.

### Statistical analysis

#### Calculation of the sample

The study was designed to determine the ultrasound criteria for hemidiaphragm paralysis. Furthermore, in patients with hemidiaphragm paralysis, the non-paralyzed side was studied and compared with ultrasound results in subjects with normal lung function.

Calculation of the number of patients to be included was performed for comparison between patients with no pulmonary disease and patients with hemidiaphragm paralysis. In a previous study (31), it was reported that, excursions measured during quiet breathing were increased, on the healthy side of patients with hemidiaphragm paralysis compared to healthy controls, for both the left side (3.3 ± 0.9 vs. 2 ± 0.5 cm—*p* < 0.001) and the right side (3 ± 0.6 vs. 2 ± 0.5 cm—*p* < 0.001). To find differences between groups, the calculation of the sample was based on this study. For a 0.05% risk alpha and a 95% power, it was calculated that at least 26 subjects should be included for the comparison of the left hemidiaphragm excursions and 20 subjects for the right hemidiaphragm excursions.

Results of the measurements were reported as mean ± standard deviation. Statistical tests were run on Sigma Stat software (SPSS Inc., Chicago, United States). The ultrasound parameters recorded on the non-paralyzed side were compared with measurements performed in subjects without pulmonary disease. Analysis was performed using unpaired student’s *t*-test. In the event of cohorts of variables not having a normal distribution, comparisons were performed with the Mann–Whitney *U* test.

It has been suggested that a paralyzed hemidiaphragm develops atrophy (26), so the thickness measured on the paralyzed side was compared to the measurement on the other side. For this comparison a paired *t*-test was used. When the distribution was not Gaussian, a Wilcoxon test for paired data was performed.

The significance level was *p* < 0.05.

## Results

### Populations

To begin with, 106 patients with a recognized hemidiaphragm paralysis were screened. Three subjects could not be included due to significant pleural effusion in 1 case or poor image quality leading to the impossibility of measuring the diaphragm thickness in 2 cases.

In total, 103 patients (40 women, 63 men) were ultimately included in the study; The paralysis was on the right side in 39 cases (17 women and 22 men), on the left side in 64 cases (23 women, 41 men). Two patients (1 woman, 1 man) had a diagnosis of hemidiaphragm paralysis on one side (left) and a dysfunction without complete paralysis on the other side.

The context of discovery of the hemidiaphragm paralysis was surgery (cervical or thoracic surgery) in 62 cases (thoracic surgery in 59 cases and cervical surgery in 3 cases). In 13 cases patients had several thoracic surgery procedures (multiple surgeries in 13 patients), trauma in 16 cases, neuro-muscular disease in 6 cases, infectious disease in 4 cases, ablation of atrial fibrillation in 3 cases and neoplasia in 2 cases. In some patients, several contributing factors could be involved such as thoracic surgery and atrial fibrillation ablation in 3 patients, a history of thoracic surgery and thoracic neoplasia in 1 patient, a history of chest trauma and thoracic neoplasia in 1 patient and thoracic neoplasia and atrial fibrillation ablation in 1 patient. In 5 cases, no predisposing factor for hemidiaphragm paralysis was identified and etiology remained unknown.

Table 1 reports the characteristics of the population studied. In patients with hemidiaphragm paralysis, the pulmonary function tests were consistent with a restrictive pattern.

**Table 1:** Characteristics of the population studied.

	Patients WPD	Subjects with HP	*p*-value
Women/Men	42–68	39–64	NS
Percentage of women %	38	39	NS
Age	55 ± 14	58 ± 14	NS
Height	171 ± 9	171 ± 9	NS
Weight	74 ± 14	73 ± 16	NS
SVC (L)	4.2 ± 1	2.7 ± 0.9	<0.001
SVC (% of predicted)	101 ± 13	68 ± 16	<0.001
TLC (L)	6.1 ± 1.3	4.8 ± 1.2	<0.001
TLC (% of predicted)	99 ± 13	83 ± 15	<0.001
FVC (L)	4.1 ± 1	2.6 ± 0.9	<0.001
FVC (% of predicted)	102 ± 13	69 ± 16	<0.001
FEV _1.0_ (L)	3.2 ± 0.9	2 ± 0.7	<0.001
FEV _1.0_ (% of predicted)	99 ± 16	67 ± 16	<0.001
FEV _1.0_/FVC	78 ± 11	77 ± 10	NS

WPD, without pulmonary disease; HP, hemidiaphragm paralysis; SVC, slow vital capacity; TLC, total lung capacity; FVC, forced vital capacity; FEV_1.0_, expiratory volume in 1 s.

### Ultrasound study

In the 103 patients included, all ultrasound parameters were successfully measured. The measurement of the maximal left hemidiaphragm excursion during deep inspiration was not possible in 6 subjects without pulmonary disease out of 120 (5%).

Table 2 reports mean excursions, thicknesses, and percentages of thickening of the paralyzed hemidiaphragm.

**Table 2:** Ultrasound findings on the paralyzed side (mean ± SD).

		Right (39 patients)	Left (64 patients)
Quiet breathing	Excursion (cm)	0 ± 0.5	−0.1 ± 0.4
Voluntary sniffing	Excursion (cm)	−1.2 ± 0.5	−1.1 ± 0.4
Deep inspiration	Excursion (cm) Initial motion	−0.3 ± 0.8	−0.5 ± 0.5
	Terminal motion	1.2 ± 0.6	1.3 ± 0.9
Thickness	At end expiration (mm)	1.5 ± 0.6	1.5 ± 0.5
Inspiratory thickening	At quiet breathing (%)	−1 ± 5	−2 ± 6
Thickening fraction	At deep breathing (%)	−5 ± 9	−5 ± 11

Table 3 summarizes various profiles observed during volitional maneuvers on the paralyzed side.

**Table 3:** Ultrasound findings on the paralyzed hemidiaphragm.

	Profile	Percentage (%)
**Inspiratory thickening**		
*Quiet breathing*		
	Lack of thickening	68
	Thinning	24
	Thickening >0 and <20%	8
*Deep inspiration*		
	Thinning	53
	Lack of thickening	36
	Thickening >0 and <20%	11
**Diaphragm motion**		
*Voluntary sniffing*		
	Paradoxical motion	99
*Quiet breathing*		
	Lack of motion	64
	Paradoxical motion	26
	Delayed cranio-caudal motion	10
*Deep inspiration*		
	Paradoxical motion at the beginning followed by cranio-caudal motion	82.5
	Lack of motion at the beginning followed by cranio-caudal motion	13.5
	Lack of motion	4

#### Thickness of the paralyzed hemidiaphragm

##### End-expiratory diaphragm thickness

Patients with hemidiaphragm paralysis had a decreased thickness of paralyzed hemidiaphragm in comparison with subjects without pulmonary disease (on the right side, 1.5 ± 0.6 mm vs. 1.95 ± 0.5 mm, *p* < 0.001, on the left side 1.5 ± 0.5 mm vs. 1.8 ± 0.4 mm, *p* < 0.001). Furthermore, the paralyzed hemidiaphragm was thinner than the contralateral one:

- In patients with left hemidiaphragm paralysis: 1.5 ± 0.5 mm on the paralyzed side vs. 1.9 ± 0.4 mm on the non-paralyzed side (*p* < 0.001).
- In patients with right hemidiaphragm paralysis: 1.5 ± 0.5 mm on the paralyzed side vs. 1.7 ± 0.5 mm on the non-paralyzed side (*p* < 0.001).

Thickness of the paralyzed hemidiaphragm measured at the end of expiration was below the lower limit of normal (i.e., <1.1 in women, <1.3 in men) in 28 patients (27%). All subjects without pulmonary disease had diaphragm thickness in the normal range.

##### Diaphragm thickening during volitional maneuvers

During quiet breathing, a lack of thickening was observed in 70 patients (68%), a thinning in 25 patients (24%), and a thickening lower than 20% in 8 patients (8%). During deep inspiration, a lack of thickening was recorded in 37 patients (36%), a thinning in 55 patients (53% of cases), and a thickening lower than 20% in 11 patients. No patient had a thickening fraction larger than 20%.

#### Motion of the paralyzed hemidiaphragm

During quiet breathing, in most of cases (66, i.e., 64%), no detectable motion was observed on the paralyzed hemidiaphragm. In 27 patients (26% of cases), a paradoxical (cranial) displacement (less than 0.5 cm) was observed.

During the sniff maneuver, a paradoxical motion was recorded in all except one patient. The motion recorded appeared as a quick negative excursion measured at 1.2 cm of mean magnitude (from −0.3 to −2.4 cm).

During deep inspiration, in 84 patients (82% of cases) the motion recorded a paradoxical motion at the beginning of the inspiration followed by a reestablishment of the motion in the cranio-caudal direction. In 14 patients, a lack of motion was recorded at the beginning of deep inspiration and after a mean delay of 0.4 s a cranio-caudal excursion was observed. Lastly in 4 patients no motion of the paralyzed hemidiaphragm was recorded.

#### Motion of the hemidiaphragm on the healthy side

Tables 4, 5 reported the diaphragmatic motions recorded on the healthy side in the patients suffering from hemidiaphragm paralysis in comparison with subjects without pulmonary disease. Statistical analysis reported that excursions measured during quiet breathing and voluntary sniffing, as well as thickening fraction and inspiratory thickening, were significantly increased on the healthy side in patients with hemidiaphragm paralysis compared to those measured on the same side in subjects without pulmonary disease.

**Table 4:** Comparison of ultrasound markers of left hemidiaphragm between subjects without pulmonary disease (WPD) and patients with right hemidiaphragm paralysis (RHP).

	Subjects (WPD)	Patients (RHP)	*p*-value
Men, women (% of women)	68 − 42 (38%)	22 − 17 (44%)	NS
Age (years)	55 ± 14	59 ± 16	NS
Height (cm)	171 ± 9	170 ± 10	NS
Weight (kg)	74 ± 14	71 ± 13	NS
Inspiratory excursion (QB) (cm)	2 ± 0.5	3.5 ± 0.9	<0.001
Voluntary sniffing (cm)	2.5 ± 0.6	3.7 ± 0.8	<0.001
Inspiratory excursion (DI) (cm)	5.7 ± 1.1	6 ± 1.2	NS
Thickness at end expiration (mm)	1.8 ± 0.4	1.7 ± 0.5	NS
Inspiratory thickening (QB) %	32 ± 16	61 ± 30	<0.001
Thickening fraction (DI) %	116 ± 33	134 ± 53	<0.02

QB, quiet breathing; DI, deep inspiration.

**Table 5:** Comparison of ultrasound markers of right hemidiaphragm between subjects without pulmonary disease (WPD) and patients with left hemidiaphragm paralysis (LHP).

	Subjects (WPD)	Patients (LHP)	*p*-value
Men, women (% of women)	68 − 42 (38%)	41 − 23 (36%)	NS
Age (years)	55 ± 14	58 ± 13	NS
Height (cm)	171 ± 9	171 ± 9	NS
Weight (kg)	74 ± 14	75 ± 17	NS
Inspiratory excursion (QB) (cm)	2 ± 0.5	3 ± 0.9	<0.001
Voluntary sniffing (cm)	2.5 ± 0.6	3.1 ± 1	<0.001
Inspiratory excursion (DI) (cm)	5.5 ± 1	5.7 ± 1.4	NS
Thickness at end expiration (mm)	1.95 ± 0.5	1.9 ± 0.4	NS
Inspiratory thickening (QB) %	35 ± 17	68 ± 48	<0.001
Thickening fraction (DI) %	116 ± 45	134 ± 53	<0.02

QB, quiet breathing; DI, deep inspiration.

This hyperactivity during quiet breathing led to an excursion of the right hemidiaphragm larger than the upper limit of normal (i.e., 2.8 cm for men and 2.5 cm for women) in 70% of patients with left hemidiaphragm paralysis. In patients suffering from right hemidiaphragm paralysis, an increased in the excursion of the left hemidiaphragm during quiet breathing (i.e., larger than 3 cm for men and 2.5 cm for women) was recorded in 77% of cases.

### Follow-up

In patients with hemidiaphragm paralysis, a follow-up was performed by the same team in 61 patients (59%), 1 to 6 months after the first examination. This subsequent ultrasound study confirmed the hemidiaphragm paralysis in these patients.

## Discussion

This study reports important ultrasound markers for the diagnosis of hemidiaphragm paralysis and highlights the need to combine M-mode and B-mode ultrasound. Some ultrasound profiles must be recognized to reduce the risk of misinterpretation.

The study of inspiratory thickening is a useful procedure for detecting hemidiaphragm paralysis. A lack of inspiratory thickening or thinning is commonly observed (92%) during quiet breathing. Nevertheless, it has been reported that inspiratory thickening may be nil during resting breathing in some healthy subjects (38). To avoid the risk of false positive, it is important to measure thickening during deep inspiration. During this maneuver, in our population and as previously reported by Gottesmann and McCool (26), thickening fraction was less than 20% in all patients. In most patients, thinning (in 53% of cases) or no change in thickness (in 36% of cases) was observed.

It can be noted that diaphragmatic thickness at its rest position i.e., as measured at end-expiration on the paralyzed side was decreased compared both to the other side and to subjects with normal pulmonary function. Nevertheless, signs of atrophy were infrequent as 72% of patients had thickness above the lower limit of normal (i.e., >1.1 in women, >1.3 in men) (33). It has been previously reported that the diaphragm can be thin in some subjects who have a normal diaphragm and suffering from generalized muscle wasting or in small-size individuals (26, 39, 40). Consequently, thickness alone cannot be used to detect diaphragm paralysis.

Measurement of diaphragmatic thickness and thickening may be difficult in some patients; Poor acoustic window was reported in 2–10% of subjects (23, 41). In our population, the measurement failed in 2 out of 106 patients, which led to the non-inclusion of these subjects in the study. In intensive care unit, failure to measure diaphragmatic thickness at end-expiration can reach 5% on the right side and 15% on the left side (42). Therefore, complementary analysis of hemidiaphragm excursion is important to strengthen the results of thickness and thickening measurement, especially when the examination is of poor quality.

As previously published (32), the study of diaphragmatic movement during breathing, at rest and sniffing is easy to achieve and the percentage of failure is very low. In our population, the study of right and left diaphragmatic motions was successfully accomplished during quiet breathing and sniffing maneuver in the entire population. Furthermore, since anatomical M-mode was used, the measurement of excursion of left hemidiaphragm during deep inspiration was easier (43, 44). In our population, the measurement failure was 5% on the left side among 120 subjects without pulmonary disease. It was successfully performed in 39 patients with right hemidiaphragm paralysis.

**Figure 2:**
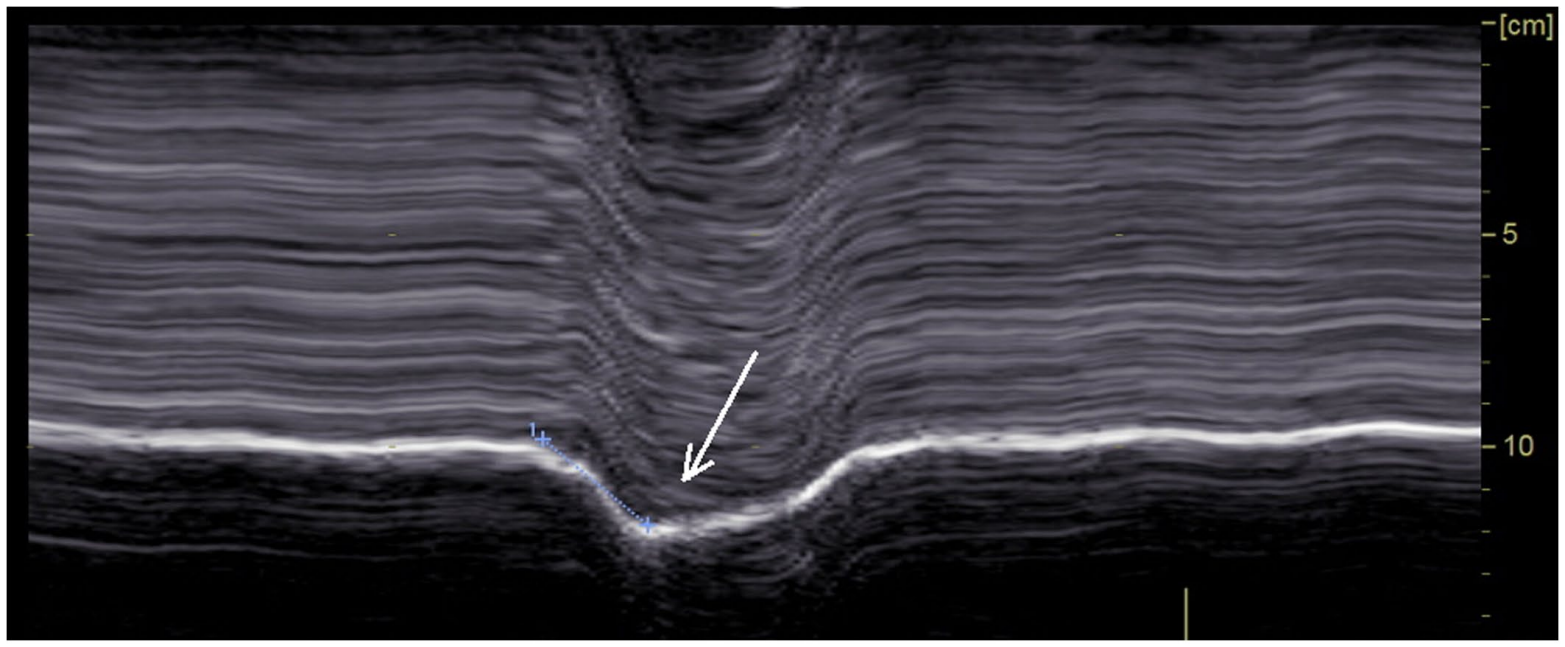
Paradoxical motion recorded during voluntary sniffing.

The analysis of diaphragm motion during sniffing, has demonstrated its interest for the diagnosis of hemidiaphragm paralysis when the recording was carried out by fluoroscopy (45) and more recently by ultrasound (24, 25). In our study, as expected, during sniffing a paradoxical motion of paralyzed hemidiaphragm was recorded (Figure 2), plausibly because of the decrease in chest pressure. This movement registered by M-mode ultrasound was a quick paradoxical displacement reaching about −1 cm (from −0.3 to −2.4 cm). In some patients, paradoxical motion was low (<0.5 cm) and no paradoxical motion was found in one case. Sniffing may be difficult to adequately perform for some patients. In addition, it was reported, using fluoroscopy, that some healthy subjects exhibited paradoxical diaphragm movement during sniffing (45). Consequently, recording of the hemidiaphragm excursion during quiet breathing and deep inspiration, may be useful as it supports the diagnosis of paralyzed hemidiaphragm.

**Figure 3:**
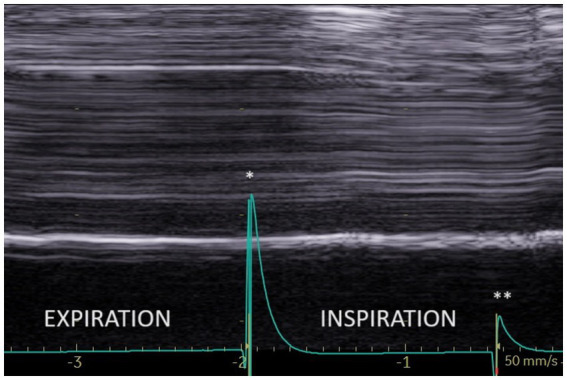
No motion during quiet breathing on the paralyzed side. ^*^Beginning of inspiration. ^**^Beginning of expiration.

**Figure 4:**
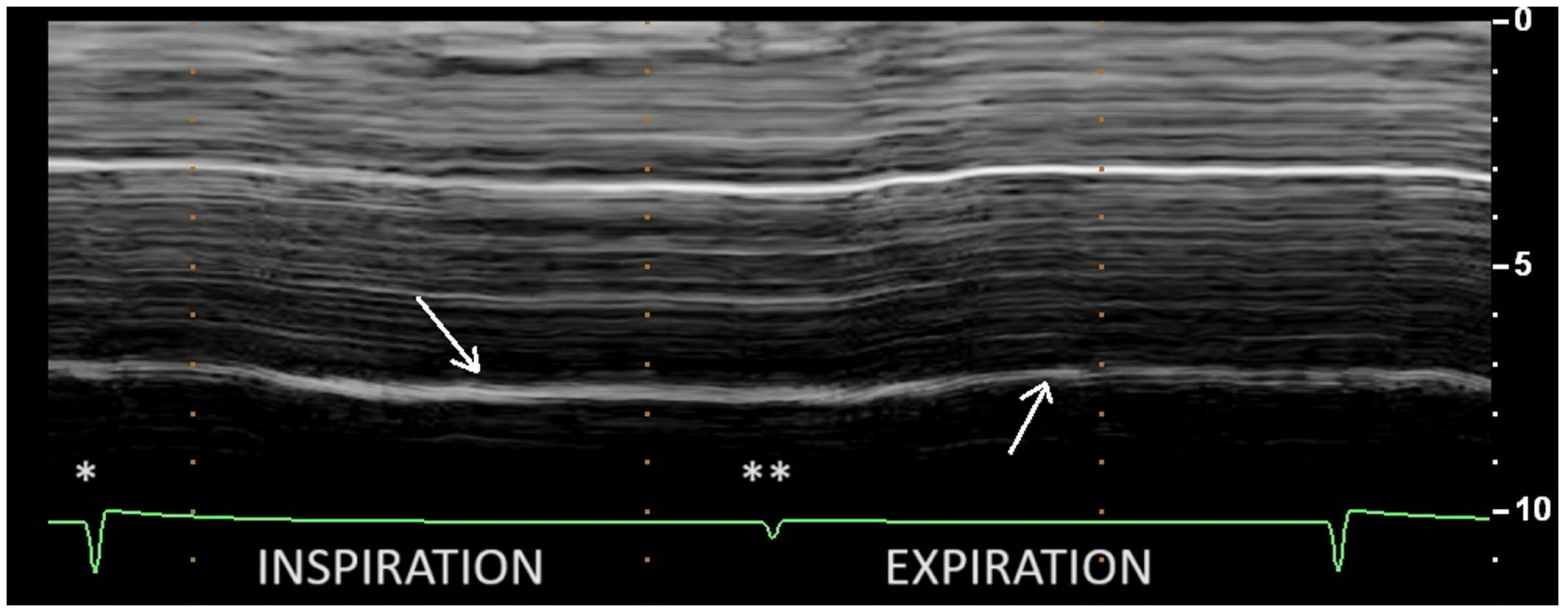
Paradoxical motion during inspiration at quiet breathing (first arrow) on the paralyzed side. ^*^Beginning of inspiration. ^**^Beginning of expiration.

During quiet breathing, on the paralyzed side, the typical no-motion picture (Figure 3) was most frequently seen (64%). In some cases (26%), paradoxical motion was detected (Figure 4). It should be noted that this motion can be misinterpreted as a normal movement since it is difficult to detect the beginning of the respiratory cycle when performing an ultrasound. It is therefore important to use a device to determine the time of the respiratory cycle corresponding to the motion.

**Figure 5:**
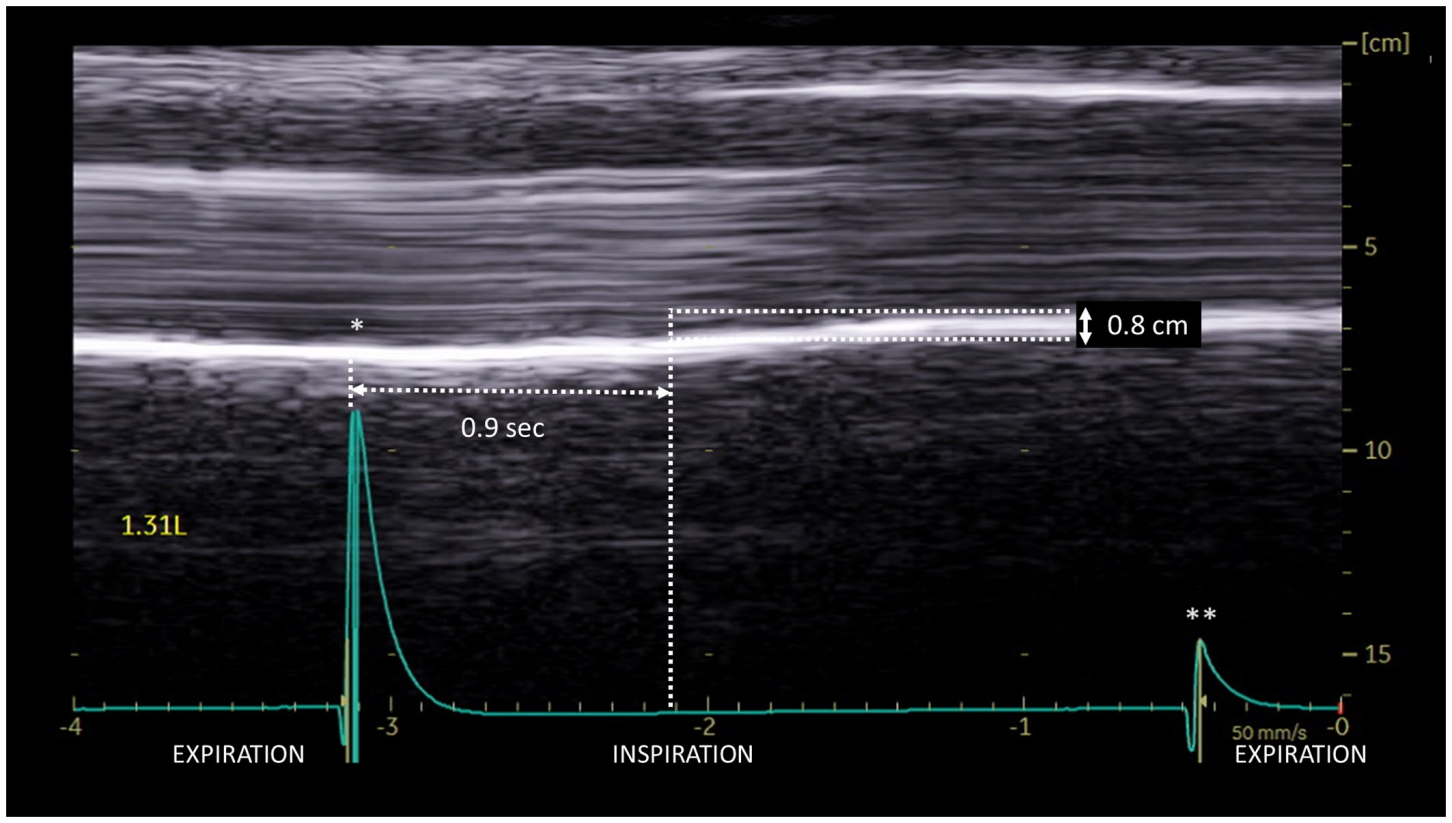
Delayed cranio-caudal excursion on the paralyzed side during inspiration (volume = 1.31 L): excursion (0.8 cm) occurred after a period of no movement at the beginning of inspiration (here 0.9 s). ^*^Beginning of inspiration. ^**^Beginning of expiration.

Another rare profile can be observed as a delayed-motion picture (Figure 5). In some subjects, during quiet breathing, a normal (cranio-caudal) excursion was seen but delayed from the onset of inspiration. This profile can also be recorded during deep inspiration (in 14% of patients). The time between the beginning of the inspiration and the caudal excursion was on average 0.4 s.

**Figure 6:**
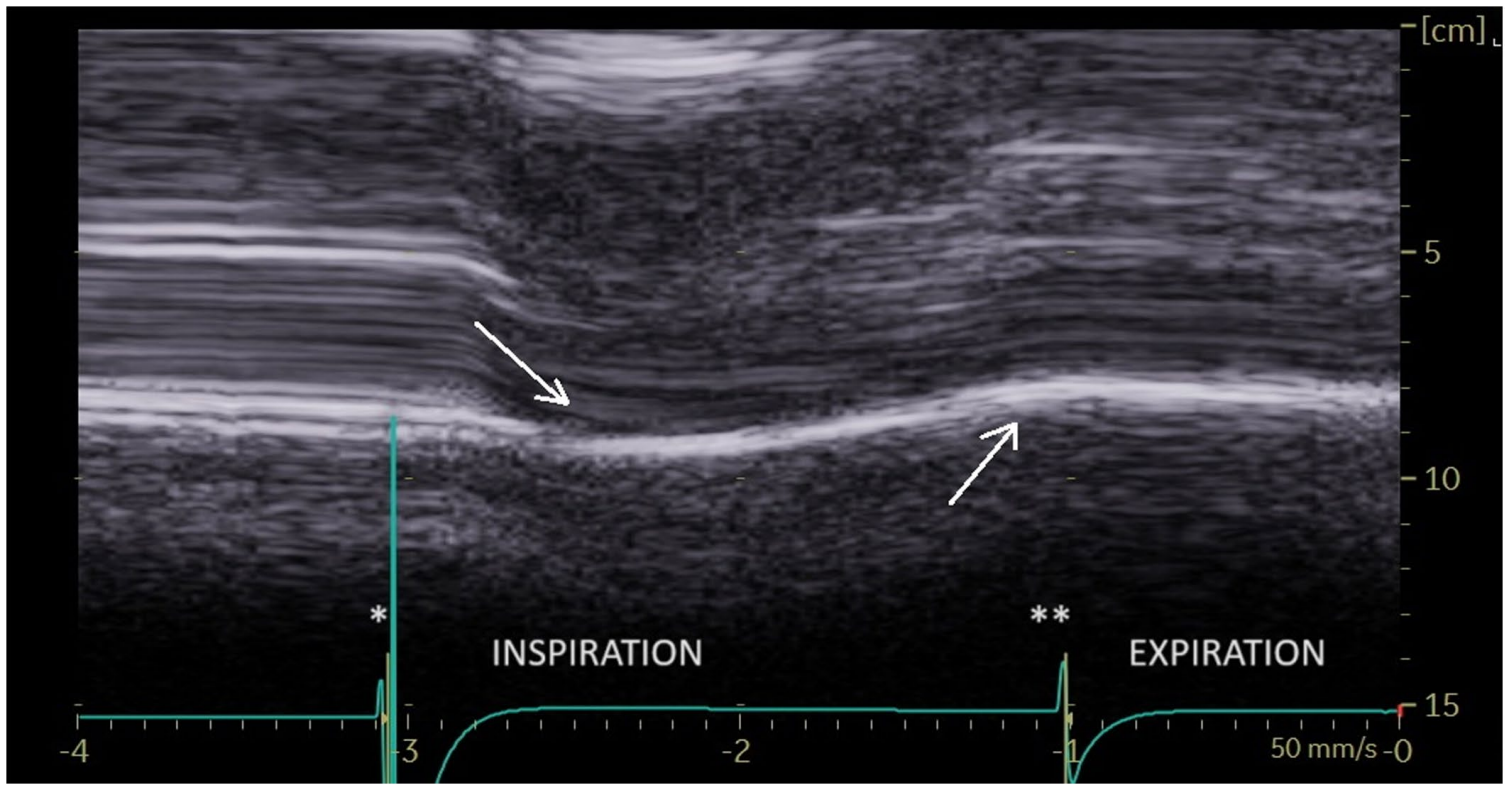
During deep breathing a biphasic movement was observed with an initial paradoxical movement (first arrow) and a terminal caudal displacement (second arrow). ^*^Beginning of inspiration. ^**^Beginning of expiration.

During deep inspiration, most patients (86%), showed a biphasic movement of the paralyzed hemidiaphragm, with an initial paradoxical cranial movement and a terminal caudal displacement (Figure 6). The caudal displacement could either be absent or reach more than 2 cm. This kinetics has previously been reported by Patel et al. (27) and Boussuges et al. (25).

The risk of misinterpretation of diaphragmatic motion analysis during deep inspiration has already been reported by studies using fluoroscopy. It was observed that during monitoring of the hemidiaphragm displacement, some patients had cranio-caudal displacement of paralyzed hemidiaphragm during inspiration, mimicking normal motion (45). The explanatory mechanism of this displacement is recognized and secondary to various factors. Relaxation of the abdominal wall could contribute to caudal displacement. Indeed, some patients may have an active contraction of the abdominal muscles during expiration to volumes below functional residual capacity. At the onset of inspiration, sudden relaxation of the abdominal muscles can lead to a caudal movement of the paralyzed diaphragm (46, 47). The role of healthy contralateral hemidiaphragm and thoracic accessory muscles has also been shown (48). The displacement of the inactive hemidiaphragm during inspiration is determined by the balance between the decrease in pleural pressure, which leads to a cranial displacement, and the force generated by the intact inspiratory muscles, which induces a caudal displacement (49). In patients with hemidiaphragm paralysis, it has been demonstrated that the neural drive to the inspiratory muscles increased (50). The strong contraction of contralateral hemidiaphragm associated with hyperactivity of the inspiratory accessory muscles expand both lungs which induce a passive caudal displacement of paralyzed hemidiaphragm.

As previously reported (51), signs of hyperactivity of contralateral hemidiaphragm were observed in our study. Indeed, inspiratory thickening, thickening fraction, excursion recorded during quiet breathing and voluntary sniffing, were increased on the healthy side in patients with hemidiaphragm paralysis compared to those measured in 120 subjects without pulmonary disease. In addition, more than 70% of patients had an excursion above normal values during quiet breathing. This result can support the diagnosis in case of suspicion of unilateral diaphragmatic dysfunction.

### Study limits and perspectives

In order to provide accurate ultrasound criteria for hemidiaphragm paralysis, we focused on patients with a recognized diagnosis. Although the included patients have been studied by various recognized methods, no method is 100% reliable for diagnosing paralysis. In addition, the distinction between complete paralysis and weakness can be difficult. The time between the ultrasound and various other examinations was as short as possible. This is important because it has been reported that functional recovery can occur in about 50% of patients within 2 years of diagnosis (52). In our study, a second ultrasound was performed in 61 patients out of 103 and the criteria for hemidiaphragm paralysis were still present in all of them.

In the patients studied, diaphragm thickness was decreased on the paralyzed side as compared to the healthy side. However, the thickness was rarely below the lower limit of normal (27% of cases). It was suggested that measuring the diaphragm thickness could be useful in assessing the date of the lesion (26). An old paralysis leads to atrophy whereas the diaphragm may have a normal thickness after a recent paralysis. In our population, it was often difficult to date the onset of diaphragm dysfunction because many patients had several predisposing factors appearing over several years. Further studies would be interesting to assess the evolution of diaphragm thickness after paralysis. This would be useful to determine the age of paralysis and specify the criteria for predicting functional recovery.

## Conclusion

Hemidiaphragm paralysis can remain unrecognized for a long time (27). It is important to detect this paralysis by ultrasound after risky circumstances such as after trauma or after cervical and thoracic surgeries which accounted for 76% of contributing factors in our study. In critically ill patients and subjects with comorbidities, diagnosis is important because hemidiaphragm paralysis can contribute to respiratory failure. In addition, it has been reported that persistent diaphragm dysfunction in patients undergoing elective cardiac surgery was associated with adverse respiratory outcomes (53). In unilateral diaphragm paralysis, inspiratory muscle training improves clinical condition through strengthening of healthy hemidiaphragm and accessory inspiratory muscles (54). In patients with no recovery and persistent disabling respiratory disorders, diaphragm plication can be proposed (55). For accurate analysis of the diaphragm function, the ultrasound study should include thickening and excursion measurements. The different profiles reported by our study are useful to avoid misinterpretation because passive excursions can mimic normal motion. In addition, the use of a device that determines the breathing phase can aid in detecting paradoxical and passive movements.

## Data availability statement

The raw data supporting the conclusions of this article will be made available by the authors, without undue reservation.

## Ethics statement

This study fell within the French legal framework of non-interventional research and was classified as non-involving human subjects. Ethical approval was not required for the analysis of data collected during standard clinical care. In the medical consultation unit, all patients received a notice of information and non-objection in accordance with French law. The written informed consent to participate in this study was not required from the participants or the participants’ legal guardians/next of kin in accordance with the national legislation and the institutional requirement.

## Author contributions

AB: Writing – original draft, Writing – review & editing, Conceptualization, Investigation, Methodology. AF: Writing – review & editing, Investigation. ML: Writing – review & editing. GB: Investigation, Writing – review & editing. AM: Investigation, Writing – review & editing. LZ: Methodology, Writing – review & editing. SD: Methodology, Writing – review & editing. MG: Investigation, Methodology, Writing – review & editing. HD: Methodology, Writing – review & editing. FB: Writing – original draft.
